# Time change in the distribution of physical activity and its correlates among retired older Swedish adults: a repeated cross-sectional study from a national survey

**DOI:** 10.1186/s12889-022-14554-2

**Published:** 2022-11-09

**Authors:** Bertil Vilhelmson, Eva Thulin, Erik Elldér

**Affiliations:** Department of Economy and Society, University of Gothenburg, PO Box 625, 405 30 Gothenburg, SE Sweden

**Keywords:** Time-use diaries, Daily activity domains, MVPA, MET, Ageing, Time-use epidemiology

## Abstract

**Background:**

Understanding how older adults spend time in moderate- to vigorous-intensity physical activity (MVPA) is crucial to understanding healthy ageing. This study connects 24-h time-use diary records of the daily activities of a sample of Swedish older adults to energy intensities. The aim was to: i) estimate the prevalence of Swedish older adults (aged 65–84 years) who achieved recommended daily levels of physical activity; ii) identify what domains of everyday life contribute to MVPA; and iii) explore socio-demographic factors affecting rates of active living.

**Methods:**

We draw on two Swedish nationally representative samples of time-use diary data from 2000/2001 and 2010/2011. Data covering the duration of all activities performed over two days were combined with activity-intensity information (metabolic equivalent of task [MET] values) to estimate the energy expenditure (MET min) originating from MVPA.

**Results:**

Results indicate that 94.1% of Swedish older adults achieved the WHO-recommended minimum level of daily MVPA in 2010/2011; the share remained unchanged over the period. MVPA performed in natural environments (24.2%), during housework (22.8%), and on everyday walks in one’s local area (18.1%) were dominant domains contributing to energy expenditure. Home maintenance and repairs (8.8%), active transport (9.9%), and physical exercise (8.2%) contributed to a lesser extent. In 2000/2001, total MVPA energy expenditure was associated with gender, housing, living region, and disability; in 2010/2011, except for disability, these associations were no longer significant.

**Conclusions:**

The high proportion of older adults who achieved the recommended level of MVPA, their allocation of MVPA time to diverse domains, and the reduced social distribution over time suggest that elderly people increasingly find their own paths to everyday physical activity. This indicates a need to promote MVPA not only in established ways, such as prescribed training programmes. The importance of active physical activities in natural environments, and of regular walks in the vicinity of home, indicates a need to incorporate healthy ageing considerations in wider urban and regional planning, for example, to increase access to natural environments and urban walkability. Also, older adults’ involvement in household chores, maintenance and repairs, and active transport extends responsibility to new policy areas.

**Supplementary Information:**

The online version contains supplementary material available at 10.1186/s12889-022-14554-2.

## Introduction

Numerous studies document the importance of physical activity and associated energy expenditure for maintaining and promoting individual health and well-being even in old age [1–3]. Even so, studies of all activities performed by older populations throughout the 24 h of the day are rare [4]. This also applies to studies of changes over time when new generations retire. With a few important exceptions [5–7], previous time-use research has concentrated on *narrower* ranges of activities performed during leisure time and outside the home [8, 9], such as organized sports and gym exercising [10], walking and jogging [11], and outdoor adventure activities in nature [12]. Such activities are also at the fore of policy and interventions, and in the Nordic countries, for example, physical activity can be prescribed by healthcare services (“physical activity on prescription”) [13]. These activities are often contrasted to what are considered ordinary in-home sedentary pursuits. Still, recent research indicates the positive health contribution of activities performed in other domains of everyday life, both inside and outside the home. For example, studies demonstrate the positive health effects of ordinary housework [14], home gardening [15], and walking and cycling for transportation [16]. Spending time in these domains also intersects with other important aspects of sustainable living, for example, walking and biking can replace car use [17] and spending time in nature and gardening are associated with better mental health and subjective wellbeing [18].

This points to a research need to widen the analysis of physical activity and consider how older people allocate their time to various activities in different life domains throughout the day. Time-use surveys (TUSs) facilitate such information gathering at a detailed level of activity and duration, although TUSs lack information on the intensity and energy expenditure of specific activities.

To overcome this, and in view of the important methodological work of Tudor-Locke et al. [19], Liangruenrom et al. [20] and Harms et al. [21] connecting the International Classification of Activities for Time-Use Statistics with the Compendium of Physical Activities [22], we link time spent in different activities to their energy-intensity characteristics in terms of metabolic equivalent of task (MET) values. This allows estimates of physical activity energy expenditure over the 24 h of the day. We can thus assess how behaviour may influence daily physical activity energy expenditure and ultimately health and wellbeing. From a policy measure perspective, an important point of departure for this is the WHO [3] recommendation that older adults (65 + years old) should engage in at least 150–300 min of moderate-intensity aerobic physical activity (MPA), at least 75–150 min of vigorous-intensity aerobic physical activity (VPA), or an equivalent combination of moderate- and vigorous-intensity activity (MVPA) throughout the week.

This study aims to explore the full range and distribution of time spent on active physical activities among the older population in Sweden also taking energy intensity into account. *(i)* We estimate the fraction of the population that achieves sufficient levels of MVPA energy expenditure during the day and whether this fraction changes over time. *(ii)* Furthermore, we identify what particular domains of daily activities contribute to sufficient levels of MVPA and *(iii)* how energy expenditure is associated with various socio-demographic factors at two points in time over a ten-year period.

## Method

We conducted a repeated cross-sectional analysis of the Swedish Time Use Survey from 2000/2001 and 2010/11. Respondents were aged 65 to 84 years. Data covering the duration of all activities performed over two days were combined with activity-intensity information (MET values) to estimate the total energy expenditure (MET min) originating from MVPA. Multiple regression analysis was used to examine socio-demographic factors associated with MVPA, in total and by domain.

### Data

The data were described and assessed in a previous publication [4]. Time-use data were drawn from the two most recent cross-sectional national TUSs carried out by Statistics Sweden in 2000/2001 and 2010/2011. The participants were identified and recruited based on random samples drawn from the Swedish Register of the Total Population (RTB). They were contacted through an introductory letter which also contained the diary to keep during the two measurement days. Then the participants were contacted through home visits (in the 2000/01 survey) or telephone (in the 2010/11 survey). In the surveys, each respondent kept a time-use diary for two discrete days, one weekday and one Saturday or Sunday, chosen in a random week during the year of measurement. In the diary, the day was divided into 10-min periods. For each period, respondents described in their own words what they were primarily doing. The information was then coded into over 120 primary activities typical of everyday life. Coding was performed by trained staff at Statistics Sweden, using an established, detailed coding instruction manual to avoid inter-coder discrepancies. The subsamples used here were representative of all retired older adults 65–84 years old registered in Sweden at the survey times and comprised a total of 288 and 509 individuals in 2000/2001 and 2010/2011, respectively.

The response rate of the survey as regards the population aged 65–84 years old (retired and non-retired) was 46% in 2000/2001 (*n* = 976) and 44% in 2010/2011 (*n* = 1308), potentially creating biased results. However, overall comparisons between the samples and the total population regarding known background factors indicated only small differences. Single people were slightly overrepresented in the 65–74-year age group in both 2000/2001 and 2010/11. In the 75–84-year age group, single people were slightly overrepresented in 2000/2001, but underrepresented in 2010/2011.

The study received ethical clearance from the Swedish Ethical Review Authority and from Statistics Sweden to access anonymized TUS microdata on individuals.

### Assigning energy intensities and calculating total MVPA energy expenditures

To calculate total energy expenditure, primary activities were each assigned an energy-intensity value based on the Compendium of Physical Activities [22] coded in MET values. Matching was done using the International Classification of Activities for Time-Use Statistics, enabling data to be classified into MVPA categories [19, 20], a procedure based on validated methods, ensuring that time-use data are more valid for non-occupational physical activity (PA) monitoring than are traditional self-report methods [6, 21, 23]. As concluded by Harms et al. [21], the uninterrupted and sequential recording used in time-use diaries covering all the activities in which respondents engage over the 24 h of the day makes it difficult for respondents to manipulate subsequent activities (e.g. substituting watching TV for taking a walk), which reduces social desirability bias and measurement error. Van der Ploeg et al. [23] found fairly high correlations between physical activity using self-reported time use diaries and accelerometer data (Spearman correlations of 0.45–0.69 for nonoccupational MVPA).

Based on current WHO guidelines [3], moderate-intensity aerobic physical activity (MPA) is defined as 3.0–5.99 METs and vigorous-intensity aerobic physical activity (VPA) as ≥ 6.0 METs, although the validity parameters are not available in the literature [24]. In our time-use dataset, 19 primary activities met the ≥ 3-MET condition, see Table 1. Since the Swedish TUS registers activities in ten-minute blocks, this ensured that MVPA was performed for a certain minimum time as a cohesive activity.

**Table 1:** Primary activities in TUS meeting the ≥ 3-MET condition

ACTIVITY	Energy intensity (MET)
Cleaning the home	3.3
Building fires, chopping wood, fetching water	3.4
Maintaining yard and garden	4.15
Walking the dog	3
Construction work, renovating the home	3.3
Home repair and maintenance	3.3
Equipment repair and maintenance	3
Unspecified maintenance	3
Helping adults in own household	3
Walking and hiking in nature	5.05
Everyday walking (in local built-up areas)	3.5
Hunting	5
Fishing	3.5
Sports and exercise outdoors	5.8
Sports and exercise indoors	5.8
Other sports or outdoor life	5.8
Transport on foot	3.5
Transport by bicycle	7.5
Transport by moped, motorcycle	3.5

By combining data on total time use for an activity with its energy intensity, we calculated the energy expenditure for each activity in terms of MET min per day of observation/measurement. We then aggregated activity-level data to the individual level for the analysis and included only those participants aged 65–84 years, not working, who registered two days of diary data. All activities recorded in the time-use diaries constituted the total MVPA energy expenditure for two whole days.

### Thresholds

WHO [3] recommends that all older adults should undertake at least 150–300 min of moderate-intensity aerobic physical activity (MPA), at least 75–150 min of vigorous-intensity aerobic physical activity (VPA), or an equivalent combination of moderate- and vigorous-intensity activity (MVPA) throughout the *week*. For additional health benefits WHO states that older adults may increase MPA to more than 300 min, or undertake more than 150 min of VPA, or undertake an equivalent in MVPA throughout the week. Converted to total energy expenditure per day, this means that individuals should perform at least 64 MET min (*threshold 1*) and preferably at least 129 MET min (*threshold 2*). Accordingly, we distinguished three groups of older adults: those not meeting the basic WHO requirements (“*inactive*”); those meeting the basic, but not additional, requirements (“*somewhat active*”); and those meeting the requirements for additional health benefits (“*active*”). Other thresholds have been applied over the years and in different contexts (e.g., [5–7]). For example, up to 2015, in its annual health surveys, the Swedish Public Health Agency (SWPHA) applied 30 min and 60 min MVPA per day, corresponding to 90 and 180 MET min per day, respectively. In order to check for robustness in the analysis of the fraction of the population that achieves sufficient levels of MVPA, we compared the WHO thresholds with those derived from the SWPHA classification.

### Physical activity domains

In the analysis, primary MVPA was divided ad hoc among six physical activity domains of everyday life: housework, maintenance and repairs, everyday neighbourhood walks, active interaction with natural environments, physical exercise, and active transportation. These are domains that to various degrees are the focus of health research, policy, and practice. *Housework* has recently received attention for making a significant contribution to MVPA among older adults [14], a contribution often neglected when not studying the entirety of everyday life and overlooking time spent in domestic (reproductive) work and obligations. For the same reason, we distinguish other household-related activities included in the *maintenance and repair* domain. Within leisure, often treated as a homogeneous domain in PA time use research, we disentangle three domains: i) *everyday walks* in one’s built-up neighbourhood, a common way to undertake daily recreation and moderate exercise; ii) organized *physical exercise and sports*, since goal-oriented working out, indoor or outdoor, is normally in focus and is prescribed to increase the amount of MVPA in order to improve an individual’s physical condition and well-being; and iii) *active pursuits in natural environments*, for example, home gardening, hiking, berry-picking, fishing and hunting. Furthermore, we distinguish the role of *active transportation*, i.e., choosing walking or biking as means of transport to reach desired activities such as shopping, meeting friends, or going to the gym.

### Statistical analysis

Using descriptive analysis, we first aggregated the individual’s energy expenditures (in MET min per day) regarding MVPA over two days. This was done to estimate the fraction of the population meeting the WHO recommendations and to explore what domains of everyday life contributed to this. We then used multivariate OLS regressions to calculate factors associated with MVPA energy expenditure (outcome variable), in total and by domain. To enable the regression analysis a few outliers (cut off set to ≥ 2,900 MET min over two days) were excluded (seven observations in 2000/2001 and 10 in 2010/2011), resulting in acceptable levels of skewness and kurtosis. High numbers of zero values, typical of time-use data, were present in the sample. Usually tobit models are used to manage this condition. However, as argued by Foster and Kalenkoski [25] and Stewart [26], evidence suggests that, in this case, OLS estimates would be similar to those obtained from tobit models; moreover, OLS estimates would be also unbiased and robust to several critical assumptions about the relationship between the factors of the model and the probability of undertaking an activity.

The following were used as covariates: age, gender, household composition, income, education, housing, living region, car access, Internet access, and need for assistance (see Table 2 for subcategories). Need for assistance served as a proxy for disability and was measured as whether the respondent frequently or only occasionally needed help from others in, for example, cooking, cleaning, shopping, and washing.

## Results

### Background data

Table 2 presents the weighted participant characteristics. Briefly, major changes occurred between 2000/2001 and 2010/2011 due to the cohorts and period under study. The shares of single people, people with low education, and people needing assistance decreased, while the shares of people with Internet access, with car access, living in urban areas, and living in single-family houses increased.

**Table 2:** Characteristics of the survey sample

		2000/2001	2010/2011	Diff
		*n* = 288	*n* = 509	
Age (65, 66 … 84 years)	Mean (SD)	73.3 (5.80)	73.0 (5.58)	–0.4
Age category	65–74 yrs	56.7	59	2.4
	75–84 yrs	43	41	–2.4
Sex	female	51.7	55.6	–0.8
	male	48.3	44.4	0.8
Household composition	single	51.7	34.4	–17.2
	cohabiting	48.3	65.3	17.2
Income	lower third	44.9	40	–5.1
	middle third	32	35.4	3.7
	higher third	23.1	24.6	1.4
Education	primary	61.7	31.2	–30.4
	secondary	24.1	43.6	18.9
	university	14.2	25.2	11.1
Housing	single-family	54.3	59.1	4.8
	apartment	45.7	41	–4.8
Living region	urban	61.4	69.7	8.2
	small town/rural	38.6	30.1	–8.2
Car access in household	yes	73.2	84.3	11.1
Internet access in household	yes	15.2	67.5	52.3
Assistance needed	yes	26.9	20.9	–5.8

Weighted to population

### Change over time

Table 3 shows that MVPA in terms of total energy expenditure per person and day on average increased from 459.7 MET min to 480.8 MET min between 2000/2001 and 2010/2011 (statistical significance, *p* < 0.05), far above threshold 1 (64 MET min) and threshold 2 (129 MET min), while the median increased from 372.0 to 429.5 MET min. The distribution (percentiles) as a whole indicates a generational shift towards higher energy expenditure among the majority of middle-performing older people, while the proportion of inactive people remained unchanged and the high-performing proportion decreased slightly. This indicates a shift in which the active part of the population has become increasingly physically active in energy terms.

**Table 3:** Descriptives for total MVPA energy expenditure (MET minutes per day)

		2000/2001	2010/2011
Mean		459.7	480.8
95% CI for mean	Lower bound	459.0	480.3
	Upper bound	460.5	481.3
Median		372.0	429.5
SD		332.2	299.1
Min/max		0/1455	0/1390
SE		0.38	0.26
Percentiles	5	33	38
	10	83	116
	25	201	245
	50	372	430
	75	671	671
	90	950	906
	95	1176	1056

Weighted to population

### How many reach the thresholds?

Table 4 shows that 6.5% of the population of pensioners aged 65–84 years did not reach the WHO-recommended threshold 1 for physical activity in 2000/2001. Ten years later, in 2010/2011, the share remained unchanged at 6.2%. However, the proportion of more active pensioners, i.e., those reaching WHO threshold 2, increased slightly from 87.2% in 2000/2001 to 88.7% in 2010/2011. The change over time became more evident when the requirements for daily MVPA were tightened, for example, stipulating at least 30 or 60 min MVPA per day. It was then found that the proportion of inactive pensioners decreased over time, from 10.8% to 7.6%, while the proportion of more active increased from 78.9% to 84.0%.

**Table 4:** MVPA energy expenditure by groups that meet WHO and SWPHA criteria

		2000/2001	2010/2011	Diff
		Percent	Percent	
*WHO criteria*				
Below threshold 1	Inactive, 0–64 MET min per day	6.5	5.9	–0.6
Threshold 1	Somewhat active, 64–129 MET min per day	6.2	5.4	–0.8
Threshold 2	Active, ≥ 129 MET min per day	87.2	88.7	1.5
	Total	100	100	
*SWPHA health survey criteria*				
Below threshold 1	0–90 MET min per day	10.8	7.6	–3.2
Threshold 1	90–180 MET min per day	10.4	8.4	–2.0
Threshold 2	> 180 MET min per day	78.9	84.0	5.1
	Total	100	100	

Weighted to population

### Activity domains contribution to MVPA energy expenditure

Table 5 identifies to what extents different domains of everyday life contributed to total MVPA energy expenditure per day. It shows that in 2010/2011 active pursuits in natural environments (24.2%), housework (22.8%), and everyday walks (18.1%) were dominant sources of MVPA, while maintenance and repairs (8.8%), active transport (9.9%), and physical exercise (8.2%) were less important. The distribution remained largely stable over the studied period as no significant changes occurred.

**Table 5:** Activity domains contribution to total MVPA energy expenditure per day

	2000/2001					2010/2011					DIFF	
	*n* = 288					*n* = 509						
	Mean	SD	Min	Max	Share	Mean	SD	Min	Max	Share	Mean	Share
Housework	95.0	108.0	0	1056	20.7%	109.8	118.8	0	660	22.8%	14.8	2.2%
Maintenance and repairs at home	38.9	125.9	0	968	8.5%	42.5	108.7	0	825	8.8%	3.7	0.4%
Regular walks	83.1	117.4	0	710	18.1%	87.0	123.3	0	665	18.1%	3.9	0.0%
Active pursuits in natural environments	101.8	165.8	0	955	22.1%	116.4	196.5	0	1074	24.2%	14.6	2.1%
Physical exercise	33.2	113.5	0	986	7.2%	41.4	110.7	0	899	8.6%	8.2	1.4%
Active transport	49.1	68.9	0	563	10.7%	47.5	79.3	0	613	9.9%	-1.6	-0.8%
Other	58.9	153.5	0	1044	12.8%	36.3	101.7	0	667	7.5%	-22.6	-5.3%
TOTAL MVPA	459.7				100.0%	480.8	118.8			100.0%	21.1	0.0%

Weighted to population

#### Socio-demographic associations

Table 6 shows how total MVPA energy expenditure was associated with socio-demographic background factors. In the most recent survey, in 2010/2011, the results indicate no socially differentiating associations except for need for assistance (the proxy variable for disabilities), which was associated negatively with total MVPA energy expenditure. However, ten years earlier, several significant associations were found. Setting aside the matter of assistance, men were more likely to perform MVPA than were women, as were individuals living in single-family houses (vs. in apartments) or in large cities (vs. in small towns and rural areas). Chronological age (within the current range) was not associated with total MVPA.

**Table 6:** Regression results: Total MVPA energy expenditure and sociodemographic factors

	2000/2001			2010/2011		
	B	SE	Sig	B	SE	Sig
(Constant)	1700.4	536.5	0.002	955.6	431.7	0.027
AGE (65, 66 … 84 years)	–8.2	7.0	0.241	–1.6	5.5	0.765
GENDER (female = 0, male = 1)	212.2	81.3	0.010	–23.3	59.1	0.693
HOUSEHOLD COMPOSITION (single = 0, cohabiting = 1)	31.7	85.5	0.711	53.6	65.1	0.410
INCOME, ref = lower third						
INCOME, middle third	140.0	92.0	0.129	98.4	65.8	0.135
INCOME, higher third	109.7	116.1	0.345	–70.6	79.0	0.372
EDUCATION, ref = primary						
EDUCATION, secondary	–67.2	96.8	0.488	48.6	65.5	0.458
EDUCATION, university	–105.4	125.1	0.400	90.5	78.0	0.246
HOUSING (single-family = 0, apartment = 1)	–351.3	81.9	0.000	–57.9	60.4	0.338
LIVING REGION (urban = 0, small town/rural = 1)	–155.5	80.8	0.055	–29.5	60.5	0.626
CAR ACCESS in household (no = 0, yes = 1)	–119.5	99.0	0.228	79.5	93.7	0.397
INTERNET ACCESS in household (no = 0, yes = 1)	132.8	120.2	0.270	81.8	66.7	0.221
HELP/ASSISTANCE (no = 0, yes = 1)	–184.0	89.6	0.041	–200.5	70.4	0.005
*R*^2^	0.145			0.054		

The relationships between socio-demographic factors and energy expenditure were also analysed for each domain (see Additional file 1), revealing the following significant trends:

- *Housework* was associated positively with gender and negatively with need for assistance, as women were more active in this than were men and the disabled less active than non-disabled individuals; in contrast, men were more active in *maintenance and repairs* than were women. Energy consumption decreased with increased age. People living in single-family houses were significantly more active than those living in apartments. When it comes to *regular walks*, the picture is unclear: residing in a single-family house and having access to a car were positively associated with regular walks in 2010/2011, a correlation not found in 2000/2001; instead, higher education was negatively and middle-income positively associated with *regular walks* in 2000/2001. Regarding *physical exercise*, those with secondary and university education were more active than those with primary education, as were residents in large cities compared with residents in small towns and rural areas. Ten years before there was a gender difference, with men being more inclined than women to perform physical exercise, but by 2010/2011 equalization had taken place. In 2010/2011, regarding active *pursuits in natural environments*, men were more active than women, as were middle-income earners compared with low-income earners, while living in apartments and disability were negatively associated with total MVPA. In the domain of *active transportation*, middle-income earners were more active than low-income earners, residents in multi-family housing more active than residents in single-family houses, and university-educated people more active than primary-educated people in 2010/2011; car availability and disability were negatively correlated with energy expenditure. Results were largely similar ten years earlier.

## Discussion

This study examined the association between 24-h time-use diary records of the daily activities of a sample of the Swedish older population and energy intensities in terms of metabolic equivalent of task (MET) values. This was done to estimate older adults’ moderate- to vigorous-intensity physical activity (MVPA) energy expenditure, taking all domains of daily life into account. Two sets of cross-sectional data enabled comparisons over a ten-year period, 2000/2001 to 2010/2011. Our study makes a useful contribution because previous research, with few important exceptions, applies more selective approaches, for example, focusing on specific physical activities during leisure time at one point in time.

Our findings show that a clear majority (94%) of Swedish older adults aged 65–84 years in 2010/2011 achieved a *sufficient level* of moderate- to vigorous-intensity physical activity (MVPA), in accordance with current WHO [3] recommendations. The proportion remained unchanged over the period, although the average MVPA energy expenditure increased slightly (4.6%), indicating that the inactive group, in critical need of improved physical condition, was not influenced by policies to promote activity, such as the public health authority’s general information on the benefits of MVPA and associated recommendations based on WHO guidelines, campaigns to increase walking in everyday life, and various models for prescribing physical activity through the health care services [13]. Rather, the observed increase in energy expenditure was concentrated in the middle-performing group, while the proportion of high-performing decreased slightly. However, tightening the requirements slightly (stipulating 30 min of MVPA per day instead of 21 min) increased the inactive share to 7.6% in 2010/2011, representing a 3.2% percentage points decrease over the period.

Our results diverge somewhat from those of the few previous studies of similar design and aim. Espinel et al. [5], using data from the 2006 Australian TUS, found that 85% of adults aged 65 years or more achieved sufficient levels of MVPA (defining recommended MVPA as ≥ 3 METs and time as ≥ 30 min/day). This was mostly attributable to activities in the household domain. Achieving the recommended level of MVPA showed no associations with age, education, or residential area. Spinney and Milward [7] found that only 39.6% of Canadians aged 65 years or more met the current national recommendation in 2010, with the proportion remaining stable over four national TUS cycles (1992, 1998, 2005, and 2010). However, their study applied a stricter threshold as regards recommended MVPA intensity (> 3.5 MET), while the time was in line with the WHO reference (≥ 21 min/day). Using Canadian TUSs, Spinney et al. [6] applied an enhanced active living threshold (EAL) defined as at least 60 min per day of MVPA ≥ 4.0 MET with each episode lasting at least 10 min; they found that only 32.5% of adults aged 65 years or more met the EAL threshold in 2005.

The observed discrepancies in results are partly due to methodological differences as regards the age groups considered (e.g. whether persons over 85 years are included or not) and how stipulated thresholds for MVPA energy intensity and activity duration are defined and measured. Furthermore, overall variations in cultural contexts as regards lifestyles and health conditions among the older population probably affect the allocation of time to activities in different domains of everyday life. Divergence may cast some doubts on the exactness of the WHO thresholds, indicating a need for further research and evaluation to perform sensitivity analyses (e.g., as regards cut-offs for METs and activity duration) checking for robustness. Also meriting attention is the range of MET values sometimes associated with a certain activity as recorded in time-use diaries. Movement on foot, for example, can vary in intensity depending, for example, on the speed, surface, and topography. Compendium guidelines [22] accordingly specify the intensity of walking as 2–6 MET depending on the context and characteristics of movement. Greater detail in assignment would require the specification of activities to a degree that time-use data rarely capture, which means that one must assume average conditions. In this study, we followed the Compendium’s method of assigning specific MET to aggregated, broadly defined activities. For example, we assigned general unspecified walking activity 3.5 METs, while when specified as walking the dog 3.0 METs were assigned.

As regards cultural and lifestyle specific aspects of physical activity, our study further contributes by clarifying the *domains* of everyday life from which MVPA energy expenditure derives. In contrast to the so-called SLOTH model commonly used in previous research [9, 27] – categorizing the activities of all 24 h of the day into five broad domains: sleep, leisure, occupation, transportation, and home – we propose a model that further details the domains of leisure and transportation. When specifying leisure activities in detail, the results showed that the contribution of *regular physical exercise* – despite being the prioritized professional recommendation to improve physical health and fitness – was small, accounting for only 9% of total MET min in 2010/2011. Instead, total energy expenditure mainly originated from active pursuits in *natural environments* (24%), *housework* (23%), and *everyday walks* for recreation (18%). Domains of lesser importance were, besides physical exercise, *maintenance work and repair* (9%) and *active transportation* (10%). The distribution between domains was quite similar in 2000/2001. Results confirm recent research pointing to the often neglected importance of household work [5, 14]. Findings further stress the importance of nature contact and interaction – possibly a cultural feature of Sweden [28] – as well as everyday walking activity. From a health-promoting perspective, this indicates a need to take account of public health arguments in urban and regional planning, for example, to improve elderly people’s access to green spaces, parks, and gardens and to improve the conditions for walking in urban areas.

As regards potential *social inequality*, the total daily MVPA energy expenditure in 2010/2011 showed no associations with age, gender, household composition, income, education, housing, living region, household access to a car, or access to the Internet; the only association, a negative one, was with disability. Compared with the 2000/2001 results, MVPA energy expenditure over the period became less gendered and less socio-spatially differentiated after retirement age. However, scrutinizing the different domains of everyday life, we found that the domains of housework and maintenance were significantly *gendered* in an expected or stereotypical way. Women were more engaged in housework, men in maintenance and repairs. Gender also played a significant role as regards active physical activities in the natural environment, where men were significantly more active than women. As regards *housing*, people living in single-family houses spent more time active in the natural environment than did people living in apartments, while the opposite held for regular walks in the local neighbourhood. This might reflect differences in access to nature and a compensatory relationship between the two domains of MVPA [28]. Finally, the observed ten-year period was associated with some major shifts due to cohort changes (i.e., increased car access among upcoming pensioners) and period effects (i.e., the spread of the Internet in society). Both are potentially space-transcending technologies reducing the bodily effort needed to overcome distance, entailing a risk of encouraging sedentary behaviour. Still, our overall results cannot confirm such negative relationships with daily energy expenditure, although car use is negatively associated with active transportation. As advanced by Rosenberger et al. [29], this highlights a need for future studies to further explore the dynamic relationships between MVPA and sedentary behaviour (including sleep) and light-intensity physical activity limited by the 24-h day.

A strength of this study was the use of two population-representative samples of elderly people who logged in detail all activities performed over 48 h (two days). Since there was no particular focus on health status or health behaviour in the survey, social desirability and reporting biases were avoided. There were, however, several limitations to the study. The use of a cross-sectional design precluded causal inferences. Diaries that cover two days may underreport activities that are performed less frequently, such as hiking in nature. The use of threshold values for MVPA approximately represented the physical activity intensity for older adults given the heterogeneity in fitness and health status [5, 30]. It is also reasonable to assume that severely ill and disabled individuals had difficulty participating in the survey, resulting in the underreporting of inactive persons. Lastly, the most recently survey used here is dated 2010/2011 and may therefore miss current trends and disruptions in time use. Yet, as generational changes in behaviour and health, and associated levels of MVPA, are slow-moving structural processes, we believe our results, based on data that features change between two points in time, are relevant contributions to current discussion. Regrettably, the latest TUS performed in Sweden in 2021 did not include all activities during a day, making comparisons impossible.

## Conclusion

Physical activity is a key determinant of healthy ageing and an important public health goal. This study provides information on how older adults spend their active time over the 24 h of the day, in what domains of daily life they spend most time on energy-intense activities, to what extent current health recommendations are met, and how physically intense activities are socially distributed. The results have implications for future policies and indicate a need to promote physical activity not only in traditional ways, that is, through prescribed training programmes. The importance of active pursuits in natural environments and regular walks in the vicinity of home widens the circle of responsibility for actively incorporating public health aspects in sustainable urban and regional planning, for example, to increase access to forests, green spaces, trails, parks, and gardens and to promote walking in urban transport planning. Older adults’ allocation of MVPA time to diverse domains – also involving household chores, home maintenance and repairs, and active transport – and the observed reduced social distribution of physical activity over time suggest that elderly people are increasingly finding their own paths to physical activity in everyday life. Potential supportive measures thus concern multiple policy spheres and actors.

## Supplementary Information

**Additional file 1: Table A1. **Regression results: MVPA energy expenditure andsocio-demographic factors; the domains Housework and Maintenance and repair. **TableA2**. Regression results: MVPA energy expenditure and socio-demographicfactors; the domains Everyday walks and Active pursuits in natural environments.**Table A3. **Regression results: MVPA energy expenditure andsociodemographic factors; the domains Physical exercise and Active transport.

## Data Availability

The datasets analysed during this study are not publicly available. Data were retrieved from Statistics Sweden’s platform for access to microdata (MONA). Without explicit permission from Statistics Sweden, users may not extract microdata from MONA. https://www.scb.se/en/
