# Supplementary material for: Time change in the distribution of physical activity and its correlates among retired older Swedish adults: a repeated cross-sectional study from a national survey
Source: BMC Public Health. 2022 Nov 9;22:2055. doi: 10.1186/s12889-022-14554-2 (PMC9647936; doi:10.1186/s12889-022-14554-2)
Supplement: Supplementary file 1 — Additional file 1: Table A1. Regression results: MVPA energy expenditure andsocio-demographic factors; the domains Housework and Maintenance and repair. TableA2. Regression results: MVPA energy expenditure and socio-demographicfactors; the domains Everyday walks and Active pursuits in natural environments.Table A3. Regression results: MVPA energy expenditure andsociodemographic factors; the domains Physical exercise and Active transport. [file 12889_2022_14554_MOESM1_ESM.docx]

**ADDITIONAL FILE 1**

**Table A1** Regression results: MVPA energy expenditure and socio-demographic factors; the domains Housework and Maintenance and repair

|  | HOUSEWORK | | | | | |  | MAINTENANCE AND REPAIR | | | | | |
| --- | --- | --- | --- | --- | --- | --- | --- | --- | --- | --- | --- | --- | --- |
|  | 2000/2001 | |  | 2010/2011 | |  |  | 2000/2001 | |  | 2010/2011 | |  |
|  | B | SE | Sig. | B | SE | Sig. |  | B | SE | Sig. | B | SE | Sig. |
| (Constant) | 83.7 | 190.2 | 0.660 | 459.1 | 165.1 | 0.006 |  | 437.1 | 209.7 | 0.038 | 457.9 | 162.6 | 0.005 |
| AGE (65, 66 … 84 years) | 2.4 | 2.5 | 0.330 | –2.1 | 2.1 | 0.318 |  | –4.5 | 2.7 | 0.098 | –4.6 | 2.1 | 0.027 |
| GENDER (female = 0, male = 1) | –54.4 | 28.8 | 0.060 | –115.3 | 22.6 | 0.000 |  | 87.0 | 31.8 | 0.007 | 125.0 | 22.2 | 0.000 |
| HOUSEHOLD COMPOSITION (single = 0, cohabiting = 1) | –13.3 | 30.3 | 0.662 | –27.5 | 24.9 | 0.269 |  | –15.9 | 33.4 | 0.633 | –29.7 | 24.5 | 0.226 |
| INCOME, ref = lower third |  |  |  |  |  |  |  |  |  |  |  |  |  |
| INCOME, middle third | 8.8 | 32.6 | 0.787 | –24.1 | 25.2 | 0.340 |  | 5.5 | 36.0 | 0.879 | –36.9 | 24.8 | 0.137 |
| INCOME, higher third | –0.5 | 41.2 | 0.990 | 14.6 | 30.2 | 0.630 |  | –42.9 | 45.4 | 0.345 | –5.6 | 29.7 | 0.850 |
| EDUCATION, ref = primary |  |  |  |  |  |  |  |  |  |  |  |  |  |
| EDUCATION, secondary | 8.2 | 34.3 | 0.811 | 8.2 | 25.0 | 0.745 |  | 2.9 | 37.8 | 0.940 | –2.6 | 24.7 | 0.915 |
| EDUCATION, university | 38.7 | 44.4 | 0.384 | 11.2 | 29.8 | 0.708 |  | 78.1 | 48.9 | 0.112 | –15.8 | 29.4 | 0.591 |
| HOUSING (single-family = 0, apartment = 1) | –35.6 | 29.0 | 0.221 | 0.4 | 23.1 | 0.986 |  | –81.3 | 32.0 | 0.012 | –95.6 | 22.7 | 0.000 |
| LIVING REGION (urban = 0, small town/rural = 1) | –56.1 | 28.7 | 0.051 | –37.6 | 23.1 | 0.105 |  | –28.0 | 31.6 | 0.377 | –11.1 | 22.8 | 0.626 |
| CAR ACCESS in household (no = 0, yes = 1) | –11.6 | 35.1 | 0.740 | 16.2 | 35.9 | 0.651 |  | –27.7 | 38.7 | 0.475 | –14.9 | 35.3 | 0.674 |
| INTERNET ACCESS in household (no = 0, yes = 1) | 34.6 | 42.6 | 0.417 | –3.7 | 25.5 | 0.885 |  | 80.5 | 47.0 | 0.088 | 3.4 | 25.1 | 0.893 |
| HELP/ASSISTANCE (no = 0, yes = 1) | –34.9 | 31.8 | 0.273 | –77.3 | 26.9 | 0.004 |  | –37.3 | 35.0 | 0.288 | –22.7 | 26.5 | 0.392 |
| *R*^2^ | 0.037 |  |  | 0.097 |  |  |  | 0.099 |  |  | 0.126 |  |  |

**Table A2** Regression results: MVPA energy expenditure and socio-demographic factors; the domains Everyday walks and Active pursuits in natural environments

|  | EVERYDAY WALKS | | | | | |  | ACTIVE PURSUITS IN NATURAL ENVIRONMENTS | | | | | |
| --- | --- | --- | --- | --- | --- | --- | --- | --- | --- | --- | --- | --- | --- |
|  | 2000/2001 | |  | 2010/2011 | |  |  | 2000/2001 | |  | 2010/2011 | |  |
|  | B | SE | Sig | B | SE | Sig |  | B | SE | Sig. | B | SE | Sig. |
| (Constant) | 391.1 | 207.0 | 0.060 | –56.7 | 179.9 | 0.753 |  | 148.6 | 288.5 | 0.607 | 162.0 | 279.9 | 0.563 |
| AGE (65, 66 … 84 years) | –3.1 | 2.7 | 0.256 | 1.4 | 2.3 | 0.552 |  | 1.6 | 3.8 | 0.665 | 0.4 | 3.5 | 0.920 |
| GENDER (female = 0, male = 1) | 28.2 | 31.4 | 0.370 | –22.5 | 24.6 | 0.361 |  | 14.0 | 43.7 | 0.750 | 63.3 | 38.3 | 0.099 |
| HOUSEHOLD COMPOSITION (single = 0, cohabiting = 1) | 42.0 | 33.0 | 0.204 | 15.3 | 27.1 | 0.572 |  | 12.3 | 46.0 | 0.790 | 30.2 | 42.2 | 0.474 |
| INCOME, ref = lower third |  |  |  |  |  |  |  |  |  |  |  |  |  |
| INCOME, middle third | 66.6 | 35.5 | 0.062 | 9.3 | 27.4 | 0.734 |  | 2.9 | 49.5 | 0.954 | 78.8 | 42.7 | 0.065 |
| INCOME, higher third | 56.4 | 44.8 | 0.209 | –8.6 | 32.9 | 0.793 |  | 82.5 | 62.4 | 0.187 | –102.9 | 51.2 | 0.045 |
| EDUCATION, ref = primary |  |  |  |  |  |  |  |  |  |  |  |  |  |
| EDUCATION, secondary | –12.1 | 37.3 | 0.745 | 12.3 | 27.3 | 0.652 |  | –23.4 | 52.0 | 0.653 | –30.2 | 42.4 | 0.478 |
| EDUCATION, university | –135.6 | 48.3 | 0.005 | 22.6 | 32.5 | 0.487 |  | –104.7 | 67.3 | 0.121 | –1.1 | 50.5 | 0.983 |
| HOUSING (single-family = 0, apartment = 1) | 11.9 | 31.6 | 0.706 | 58.7 | 25.2 | 0.020 |  | –222.5 | 44.0 | 0.000 | –117.9 | 39.1 | 0.003 |
| LIVING REGION (urban = 0, small town/rural = 1) | –13.1 | 31.2 | 0.675 | 18.6 | 25.2 | 0.460 |  | 17.6 | 43.5 | 0.685 | –19.5 | 39.2 | 0.620 |
| CAR ACCESS in household (no = 0, yes = 1) | –29.3 | 38.2 | 0.443 | 98.5 | 39.1 | 0.012 |  | 10.3 | 53.2 | 0.846 | 45.9 | 60.8 | 0.450 |
| INTERNET ACCESS in household (no = 0, yes = 1) | –10.4 | 46.4 | 0.822 | –4.0 | 27.8 | 0.886 |  | 2.8 | 64.6 | 0.966 | 56.8 | 43.3 | 0.190 |
| HELP/ASSISTANCE (no = 0, yes = 1) | –54.7 | 34.6 | 0.115 | 45.1 | 29.3 | 0.125 |  | 58.9 | 48.2 | 0.223 | –95.0 | 45.6 | 0.038 |
| *R*^2^ | 0.067 |  |  | 0.028 |  |  |  | 0.125 |  |  | 0.086 |  |  |

**Table A3** Regression results: MVPA energy expenditure and sociodemographic factors; the domains Physical exercise and Active transport

|  | PHYSICAL EXERCISE | | | | | |  | ACTIVE TRANSPORT | | | | | |
| --- | --- | --- | --- | --- | --- | --- | --- | --- | --- | --- | --- | --- | --- |
|  | 2000/2001 | |  | 2010/2011 | |  |  | 2000/2001 | |  | 2010/2011 | |  |
|  | B | SE | Sig. | B | SE | Sig. |  | B | SE | Sig. | B | SE | Sig. |
| (Constant) | 187.6 | 206.2 | 0.364 | –165.8 | 164.1 | 0.313 |  | 159 | 119 | 0.183 | 128.7 | 107.1 | 0.23 |
| AGE (65, 66 … 84 years) | –2.3 | 2.7 | 0.387 | 2.3 | 2.1 | 0.276 |  | 0 | 1.5 | 0.977 | –0.3 | 1.4 | 0.802 |
| GENDER (female = 0, male = 1) | 76.3 | 31.2 | 0.015 | –24.9 | 22.5 | 0.268 |  | –3.9 | 18 | 0.828 | –9.1 | 14.7 | 0.536 |
| HOUSEHOLD COMPOSITION (single = 1, cohabiting = 1) | 9.9 | 32.8 | 0.764 | 21.9 | 24.7 | 0.377 |  | –13.3 | 19 | 0.484 | –5.1 | 16.2 | 0.754 |
| INCOME, ref = lower third |  |  |  |  |  |  |  |  |  |  |  |  |  |
| INCOME, middle third | 23.8 | 35.4 | 0.501 | 11.1 | 25 | 0.657 |  | 35.7 | 20.4 | 0.082 | 42.7 | 16.3 | 0.009 |
| INCOME, higher third | 69.6 | 44.6 | 0.12 | 36.4 | 30 | 0.225 |  | –16.8 | 25.7 | 0.515 | 5.8 | 19.6 | 0.769 |
| EDUCATION, ref = primary |  |  |  |  |  |  |  |  |  |  |  |  |  |
| EDUCATION, secondary | 18.7 | 37.2 | 0.615 | 46.9 | 24.9 | 0.06 |  | –11.6 | 21.5 | 0.588 | 23.7 | 16.2 | 0.146 |
| EDUCATION, university | 17.2 | 48.1 | 0.721 | 49.6 | 29.6 | 0.095 |  | 21.5 | 27.7 | 0.439 | 40.1 | 19.3 | 0.039 |
| HOUSING (single-family = 0, apartment = 1) | 3.7 | 31.5 | 0.905 | 35.4 | 22.9 | 0.124 |  | 19.2 | 18.2 | 0.291 | 47.6 | 15 | 0.002 |
| LIVING REGION (urban = 0, small town/rural = 1) | –11.2 | 31.1 | 0.718 | 38.7 | 23 | 0.093 |  | –19.7 | 17.9 | 0.272 | –14.1 | 15 | 0.347 |
| CAR ACCESS in household (no = 0, yes = 1) | –8.2 | 38 | 0.829 | –5 | 35.6 | 0.888 |  | –62.6 | 21.9 | 0.005 | –68.1 | 23.3 | 0.004 |
| INTERNET ACCESS in household (no = 0, yes = 1) | –51.9 | 46.2 | 0.262 | 24.3 | 25.4 | 0.339 |  | 11.9 | 26.6 | 0.656 | 10.6 | 16.6 | 0.521 |
| HELP/ASSISTANCE (no = 0, yes = 1) | –19.1 | 34.5 | 0.579 | –32 | 26.7 | 0.232 |  | –57.4 | 19.9 | 0.004 | –38.5 | 17.5 | 0.028 |
| *R*^2^ | 0.056 |  |  | 0.03 |  |  |  | 0.091 |  |  | 0.089 |  |  |
